# The geometry of life: testing the scaling of whole-organism surface area and volume using sharks

**DOI:** 10.1098/rsos.242205

**Published:** 2025-06-18

**Authors:** Joel Harrison Gayford, Duncan J. Irschick, Johnson Martin, Andrew Chin, Jodie L. Rummer

**Affiliations:** ^1^ Marine Biology and Aquaculture, James Cook University, Townsville, Australia; ^2^ Shark Measurements, London, UK; ^3^ Department of Biology, University of Massachusetts, Amherst, MA, USA; ^4^ ARC Centre of Excellence for Coral Reef Studies, James Cook University, Townsville, Queensland, Australia

**Keywords:** allometry, constraint, Elasmobranchii, evolution, tissue allocation, three-dimensional imaging, isometry, 2/3 law, square cube rule, photogrammetry

## Abstract

The ratio of surface area to volume is a key biological parameter that underpins our understanding of physiology across all levels of biological organization. Surfaces control the rate of key reactions and processes operating within the body and between organisms and their environment. Our understanding of surface area to volume ratios is embedded in the 2/3 scaling law, stating that surface area scales with volume raised to a power of 0.66. However, most empirical studies of surface area and volume scaling in animals focus on individual cells or tissues. Comparatively few studies have addressed these scaling relationships among species or ontogenetic stages at the whole-organism level. This study uncovers quantitative support for the 2/3 scaling law in an interspecific dataset at the whole-organism level. We find that the scaling of surface area to volume across 54 shark species (exhibiting an approx. 19 000-fold variation in body mass) is nearly identical to the isometric prediction of the 2/3 scaling law. There is no evidence that this relationship is driven by ecological or physiological characteristics. One plausible explanation is the presence of developmental constraints on tissue allocation that could influence the range of possible surface areas or volumes at any given body size.

## 1. Introduction

The nature of biological surfaces, and how they relate to body size and volume at various levels of biological organization, is of fundamental importance to our understanding of biochemistry, physiology, ecology and evolution [1–6]. Surface areas, both between cells and tissues and between organisms and their environment, determine the rate of the key reactions and processes required for organisms to function [7]. These reactions shape the acquisition, transport and allocation of resources and energy, ultimately dictating how organisms interact with their environment. Animals have evolved a range of surface areas at different levels of biological organization as adaptations to distinct ecological niches and environmental conditions [5]. However, surface area does not evolve free of constraint; surface area is tied to body size and body volume by biophysical and geometric principles (equation (1.1); [3]).


(1.1) $$\begin{array}{rcl} \text{(a)} & \text{SA} & \propto L^{2}\\ \text{(b)} & \text{V} & \propto L^{3}\\ \text{(c)} & \text{SA} & \propto V^{2/3}. \end{array}$$

Equation (1.1): Scaling relationships between surface area (SA), volume (V) and body length (L).

Since the mid-1800s, organismal biologists have embedded their theoretical understanding of physiology and body size evolution in the concept of a 2/3 scaling ‘law’ [2,4,8–13]. The 2/3 scaling law describes the null geometric expectation of how the surface area of an object should scale relative to volume. Considering a simple three-dimensional shape such as a cube, surface area would increase proportionally to edge length raised by an exponent of two and volume would increase with an exponent of three (equation (1.1)). Thus, surface area should increase proportionally to volume raised by an exponent of 2/3 (equation 1.1). Despite the biological importance of surface area and volume, relatively few studies have empirically assessed the scaling of these measurements at the whole-organism level, and most of our theoretical understanding of the subject has been developed in the context of unicellular organisms (e.g. [3]). The few studies to address the scaling of whole-organism surface area and volume generally report adherence to the 2/3 law, even in ecologically diverse groups such as snakes (14) and insects [15]. While these studies provide valuable insight into our understanding of surface area and volume scaling, these relationships have yet to be tested empirically in many large clades. Additionally, existing studies (e.g. [14,15],) did not use phylogenetic comparative methods, meaning that phylogenetic non-independence may have influenced their results. Finally, existing studies for large animals typically estimate surface area using mathematical equations rather than empirical data (e.g. [14]). For these reasons, phylogenetic comparative studies addressing the scaling of surface area and volume from empirical data, particularly in taxa that reach large body sizes, are warranted.

Theoretical studies considering individual cells indicate that intraspecific and interspecific scaling coefficients of surface area to volume that are smaller or larger than 0.66 are expected only where selection favours shifts in the rate at which resources (e.g. water, ions, gases or energy) can be transferred across the respective surface [3]. In such cases, the surface area to volume ratio of cells can be altered through the evolution of fractal-like convolutions, geometric dissimilitude (flattening, elongation, etc), or the internalization of surfaces [3]. While these mechanisms have been described most thoroughly in unicells, they are potentially valid at higher levels of biological organization [3,16]. Examples include gas exchange tissues in vertebrates (e.g. lungs, gills), which have evolved high fractal dimensions, conveying elevated surface areas that scale with positive allometry [4]. Additionally, many invertebrates have evolved elongated or ‘hollow ball’ body forms consistent with geometric dissimilitude, potentially resulting in positive allometry of organismal surface area [3,17].

Surface area to volume ratios are critical in all animals for heat and gas exchange [18,19], meaning that surface area scaling relationships might differ among animal species subject to diverse environmental and hydrodynamic conditions, and thermophysiological requirements. However, besides unicells, biofilms and some small-bodied invertebrates [3], there is little evidence of mechanisms to alter whole-organism surface area scaling relationships in animals. For these reasons, further studies addressing the scaling of surface area and volume of whole organisms are warranted, particularly in larger-bodied animals. Emerging three-dimensional imaging techniques are increasingly being applied to megafauna [20,21] and provide a promising avenue through which such scaling relationships can be tested over substantial body size ranges, therefore overcoming previous logistical limitations.

**Figure 1.**
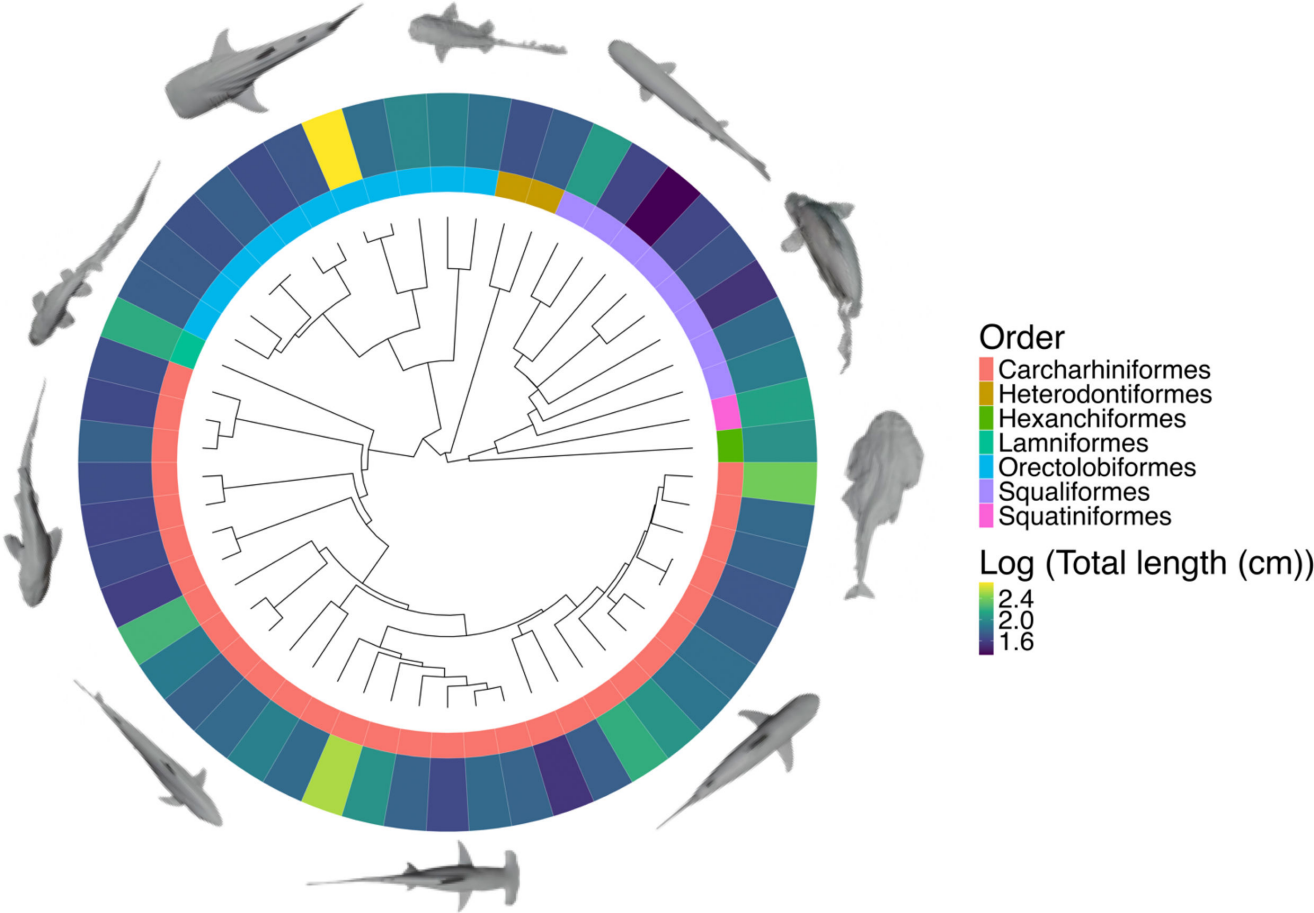
Time-calibrated maximum clade credibility (MCC) molecular phylogeny displaying the taxonomic and morphological diversity of the dataset and body size of all individuals from which meshes were produced, with select meshes displayed at the relevant positions on the phylogeny. Total length refers to the length of specific specimens used in this study, not the typical or maximum length of each species.

In this study, we find clear empirical support for the 2/3 scaling law at the whole-organism scale in sharks spanning a 16-fold increase in body length and an approximately 19 000 fold increase in body mass among 54 species (figure 1). The present study applied novel and emerging imaging techniques (summarized in [21]) to examine the scaling relationships of surface area and volume among shark species of various sizes, ecological niches and life stages within a phylogenetic framework. Sharks (Elasmobranchii: Selachii) are an ideal case study for these purposes, as they exhibit substantial variation in body size, morphology, physiology and ecological lifestyle [22]. Consequently, we might expect to observe variation in surface area to volume (SA : V) ratios at any given body size. Adult body size varies in sharks from approximately 20 cm to approximately 20 m [22], and therefore, understanding their scaling could provide valuable insight into the evolution of gigantism in marine vertebrates [23]. To measure surface area to volume ratios, we used both three-dimensional photogrammetry and computerized tomography (CT) scans of museum specimens to generate a set of accurate three-dimensional models. We discuss potential explanations for observed scaling trends and the implications of our results for the scaling of surface area and volume across animal life, which to this day remains poorly understood.

## 2. Methodology

### 2.1. Data collection

To facilitate the collection of surface area and volume data, we produced a series of anatomically accurate three-dimensional models of 54 shark species. Full body CT scans of 50 species were obtained from [24] and segmented to remove background material and internal anatomy in three-dimensional Slicer v. 5.3 [25] and converted to three-dimensional meshes. Segmented CT scans were imported into Blender v. 4.0 [26] for further processing. Specifically, we removed internal geometry not eliminated during the segmentation process (i.e. typically material relating to the gill arches and neurocranium). Additionally, we used fill and smoothing tools to ensure that meshes lacked perforations and to remove wrinkles or deformations resulting from specimen preservation. This was necessary as the CT scans used in this study come from museum specimens preserved in fluid alcohol, which typically demonstrate minor creasing, wrinkling and deformation of the dermis. It should be noted that, while some minor deformation may occur, long-term fluid preservation does not alter morphometric relationships in fishes [27], and all of these specimens were subject to preservation under identical conditions. We supplemented this initial dataset with publicly available meshes of four species produced through three-dimensional photogrammetry (DigitalLife3D, https://sketchfab.com/DigitalLife3D). While more data of this nature were available, we limited photogrammetry-mediated meshes to those made from one specific individual, as opposed to those recompiled from photographs and measurements of multiple individuals. Smoothing tools were again used in Blender here where necessary, to close all mesh perforations. Total length values for each mesh were extracted from the metadata, and we calculated both surface area and volume values in Blender using the three-dimensional Printer add-on tool.

We obtained a set of 10 000 phylogenetic trees for all extant shark species from [28], from which we inferred a maximum clade credibility (MCC) tree in R using the package ape [29]. We then pruned this MCC tree to match our dataset using the R package picante [30] and visualized this pruned tree prior to data analysis to ensure that pruning did not result in any notable changes to phylogenetic interrelationships or branch lengths.

To explore the influence of ecology on observed scaling trends, we obtained information about the ecology of each species from FishBase [31]. Specifically, species were classified as one of the following ecotypes: bathydemersal, bathypelagic, benthopelagic, demersal, pelagic or reef-associated using the ‘habitat’ variable provided on the FishBase platform.

### 2.2. Data analyses

Prior to all statistical analyses, we log10 transformed total length, surface area and volume values. Biological scaling relationships are typically expressed as an exponential relationship following the equation $Y=AX^{b}$, and hence log transformation results in a linear relationship following the equation log Y=logA+b ⋅logX, in which *b* is the scaling coefficient.

To estimate scaling relationships between surface area, volume and body size, we first fit three simple linear regression models in R: surface area approximately total length (expected scaling coefficient of 2), volume approximately total length (expected scaling coefficient of 3) and surface area approximately volume (expected scaling coefficient of 2/3). To account for phylogenetic non-independence in our dataset, we subsequently refitted each of these models using a phylogenetic generalized least squares regression (PGLS) approach in the R packages ape and nlme [29]. Our PGLS models assumed a Brownian motion covariance matrix, representing the null hypothesis for evolutionary models. We chose a Brownian motion covariance matrix over alternatives such as Early Burst or Ornstein–Uhlenbeck correlations, as it is the most commonly used null model of trait evolution (e.g. [32]), and there is no *a priori* reason to suggest alternative matrices would provide a better fit. We simply used PGLS to remove the potential confounding effects of phylogeny from our dataset [33]. While, to some extent, PGLS analyses make the previous linear models redundant, we performed both sets of analyses as GLS-based approaches cannot provide true confidence intervals or $R^{2}$-based measures of model fit, and because the phylogenetic interrelationships underpinning PGLS analyses are fundamentally hypothetical in nature. To test for deviations from isometric expectations in each of our regression models, we performed two-tailed one sample *t*-tests comparing observed slopes with slopes expected under isometry (2, 3 and 0.66 for surface area, volume and surface area to volume ratios, respectively). To account for phylogenetic uncertainty, we also performed PGLS analyses regressing surface area and volume against total length in a Bayesian framework, taking a random sample of 100 phylogenetic trees from the initial set of 10 000. These analyses were performed in the package mulTree using the method originally described in Healy *et al*. [34], using default prior settings.

To test the influence of ecology on the scaling of surface area and volume, we repeated the above (non-Bayesian) PGLS analyses and *t*-tests, considering each ecotype separately. As our dataset contains only a single bathypelagic species (*Isistius brasilensis*), it was not possible to fit a separate regression model for the bathypelagic ecotype. Rather, this species was incorporated into the pelagic ecotype for the purpose of this analysis. Again, all PGLS models assumed a Brownian motion covariance matrix. Further, to test whether the surface area to volume ratio itself (rather than the scaling relationship between surface area and volume) varied among ecotypes, we computed observed intercepts and associated confidence intervals for each of the above regression models.

To provide a visual representation of the scaling coefficients uncovered by our PGLS models, we rescaled all meshes to the size of a whale shark and remeasured surface area and volume values in Blender v. 4.0 [26].

Finally, to assess levels of phylogenetic signal underlying surface area and volume measurements, we calculated Blomberg’s K [35] for rescaled surface area and volume values. Rescaled data were used to eliminate the effects of total length, which vary dramatically among taxa in the dataset. We calculated K in the phytools package [36] and tested the significance of observed values using a simulation approach. Observed K values were compared to a null distribution of 1000 simulations obtained by randomizing tip data, to determine deviation from the expected value of zero in the absence of phylogenetic signal.

## 3. Results

**Figure 2.**
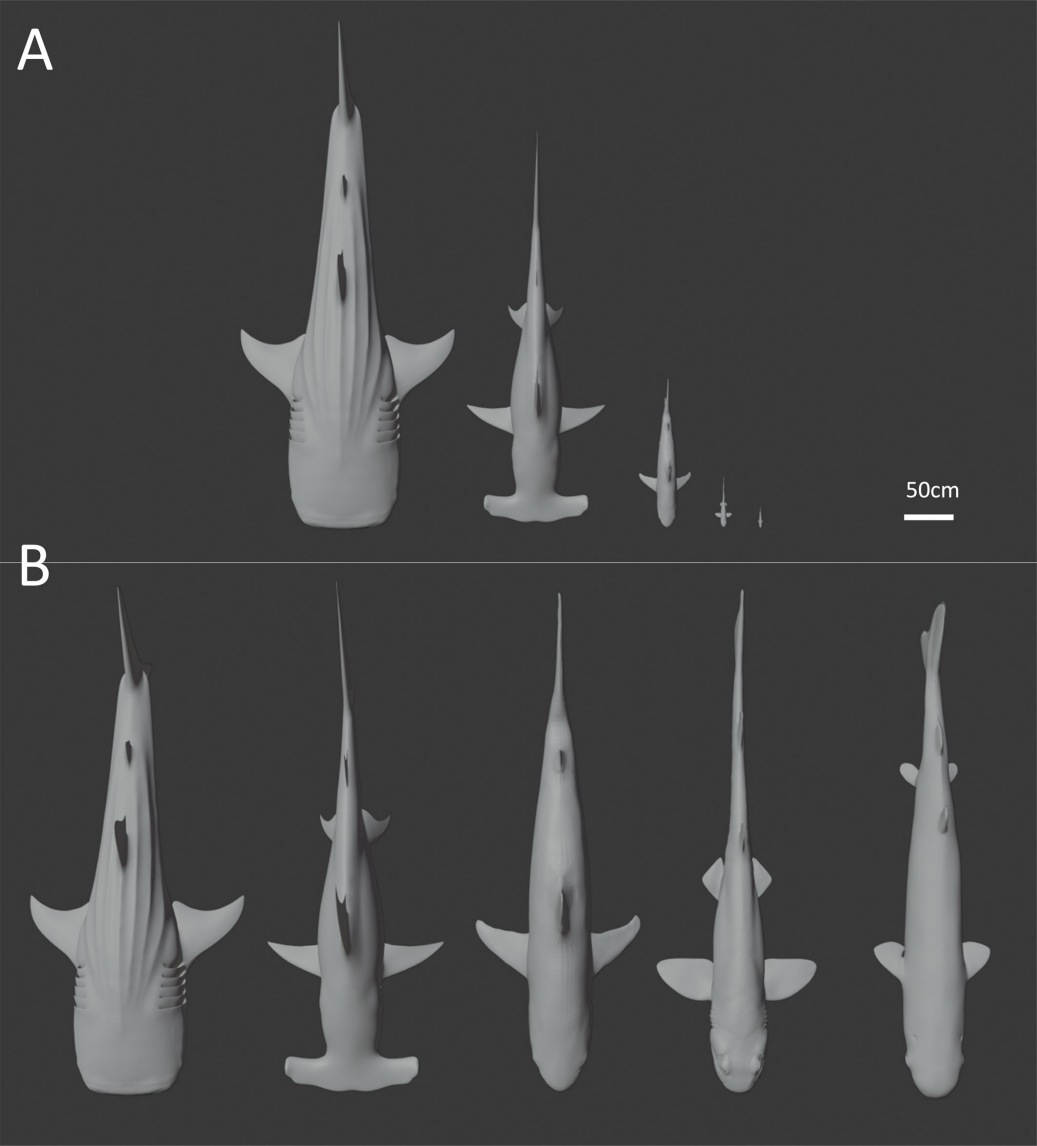
Three-dimensional models of select taxa (*Rhincodon typus*, *Sphyrna mokarran*, *Carcharhinus limbatus*, *Haploblepharus edwardsii* and *Euprotomicrus bispinatus* from left to right) to scale (A) and scaled to 500 cm total length (B) in dorsal view.

Across our dataset of 54 species, surface area varies from 125 to 100 142 cm^2^, and volume varies from 58 cm^3^ in *Euprotomicrus bispinatus* to 1 222 668 cm^3^ in *Rhincodon typus* (figure 2A). Simple linear regression models indicate that both surface area and volume scale positively allometrically with body length, and that surface area scales isometrically with volume (table 1). Each of these scaling coefficients explains a very high proportion of total variance in surface area and volume between species, with *R*^2^ values varying between 0.95 and 0.99 (table 1). After correcting for phylogenetic non-independence using PGLS regression and a Brownian motion covariance matrix, volume is still found to scale with body length with positive allometry (table 1). However, surface area scales quasi-isometrically with body length and scales with volume with slight negative allometry (table 1). Goodness of fit is not specified for these phylogenetically corrected relationships, as *R*^2^ cannot be applied to GLS-based approaches. PGLS analyses repeated over a random selection of 100 phylogenetic trees recovered mean scaling coefficients that did not differ significantly from those obtained using the MCC tree (*b*_SA_ = 2.21 +/− 0.12; *b*_*V*_ = 3.37 +/− 0.17).

**Table 1.** Scaling relationships between surface area (SA), volume (V) and body length (TL). BM refers to the Brownian motion covariance matrix applied to phylogenetically corrected models, whereas NA indicates that no phylogenetic correction was applied. LCI and UCI denote lower and upper 95% confidence intervals, respectively, for the observed slope. *t* and *p* values are reported from two-tailed, one sample *t*-tests of equivalence between expected and observed slopes.

model	phylogenetic correction	expected slope	observed slope	LCI (slope)	UCI (slope)	s.e.	$R^{2}$	*t*	*p*
logSA ~ logTL	NA	2.00	2.18	2.06	2.31	0.06	0.96	2.87	<0.01
logSA ~ logTL	BM	2.00	2.12	2.01	2.24	0.06	NA	2.10	0.04
logV ~ logTL	NA	3.00	3.28	3.09	3.48	0.11	0.95	2.86	<0.01
logV ~ logTL	BM	3.00	3.31	3.11	3.51	0.10	NA	3.04	<0.01
logSA ~ logV	NA	0.66	0.66	0.64	0.68	<0.01	0.99	0.71	0.48
logSA ~ logV	BM	0.66	0.64	0.62	0.65	<0.01	NA	3.39	<0.01

**Figure 3.**
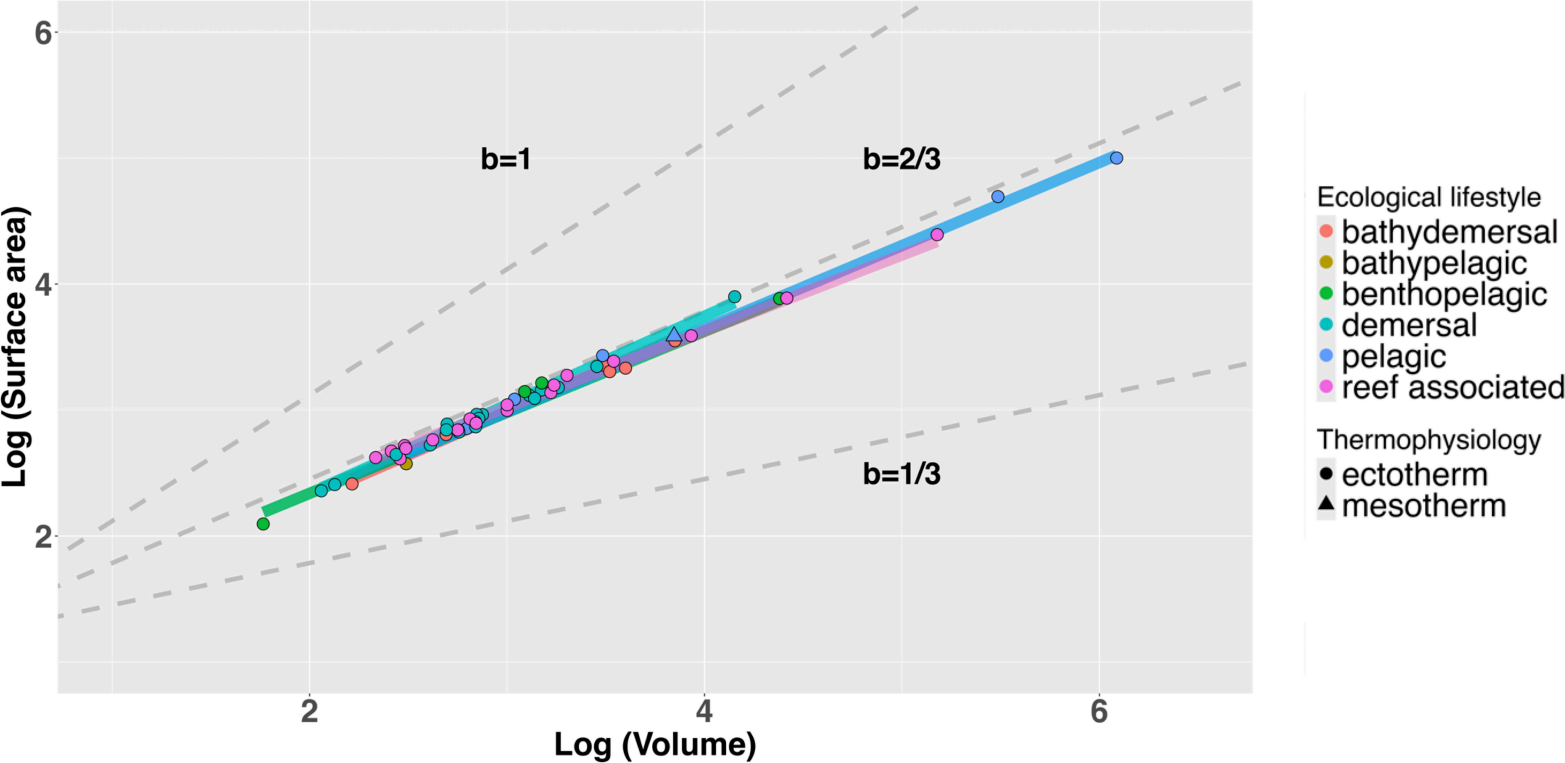
Graphical representations of phylogenetically corrected scaling relationships between body length, surface area and volume broken down by ecotype. Grey dashed lines show hypothetical relationships for scaling coefficients (where b represents the scaling coefficient). Each point corresponds to a single species. No separate analyses comparing thermophysiological strategies were performed due to the presence of only one mesothermic species in the dataset.

**Figure 4.**
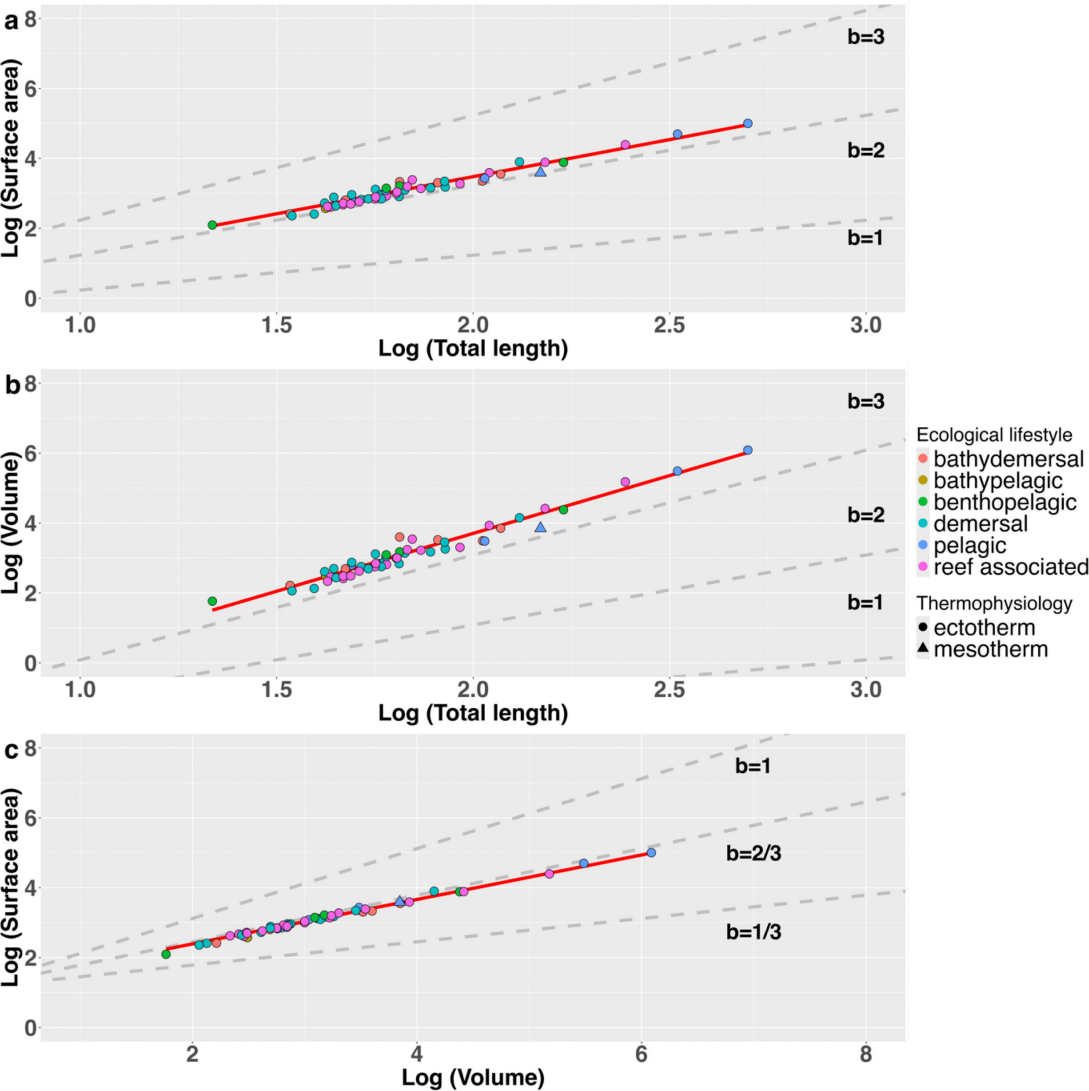
Graphical representations of phylogenetically corrected scaling relationships between body length, surface area and volume. Grey dashed lines show hypothetical relationships for scaling coefficients (where b represents the scaling coefficient). Each point corresponds to a single species. No separate analyses comparing thermophysiological strategies were performed due to the presence of only one mesothermic species in the dataset.

Some variation in the scaling of surface area to volume ratios was observed among shark ecotypes (figure 3); although, this variation was minimal (table 2). Only the reef-associated ecotype exhibited (relatively minor) statistically significant deviations from the isometric predictions of the 2/3 law, with a scaling coefficient of 0.60 (table 2). The pelagic ecotype exhibited the closest adherence to the 2/3 law, with a scaling coefficient essentially identical to the isometric prediction (figure 3; table 2). The intercepts of all ecotypes were similar, with a significant difference found only between bathydemersal and reef-associated species (table 2). The single mesothermic species in our dataset (*Lamna nasus*) did not show notable differences in surface area or volume from other species of similar body size (figures 3 and 4) or from predicted values obtained from the whole dataset (tables 1 and 2).

**Table 2.** Scaling relationships between SA and V, within individual ecotypes. For the purposes of PGLS analyses, the single bathypelagic taxon (*Isistius brasilensis*) was included in the pelagic ecotype as described in the methodology. All models assumed a Brownian motion covariance matrix applied to phylogenetically corrected models. LCI and UCI denote lower and upper 95% confidence intervals, respectively, for the observed slope and intercept values. *t* and *p* values are reported from two-tailed, one sample *t*-tests of equivalence between expected and observed slopes.

model	ecotype	expected slope	observed slope	LCI	UCI	observed intercept	LCI (intercept)	UCI (intercept)	s.e.	$R^{2}$	*t*	*p*
logSA ~ logV	bathydemersal	0.66	0.68	0.64	0.73	0.9395045	0.78922392	1.08978508	0.02	NA	0.95	0.40
logSA ~ logV	benthopelagic	0.66	0.70	0.65	0.76	1.0515043	0.6899344	1.41307411	0.03	NA	0.28	0.80
logSA ~ logV	demersal	0.66	0.64	0.53	0.76	0.9336474	0.75137952	1.11591524	<0.01	NA	1.51	0.15
logSA ~ logV	pelagic	0.66	0.66	0.63	0.69	1.0076129	0.87081368	1.14441213	0.01	NA	0.03	0.98
logSA ~ logV	reef- associated	0.66	0.60	0.58	0.63	1.210126	1.10811995	1.3121314	0.01	NA	4.94	<0.01

**Figure 5.**
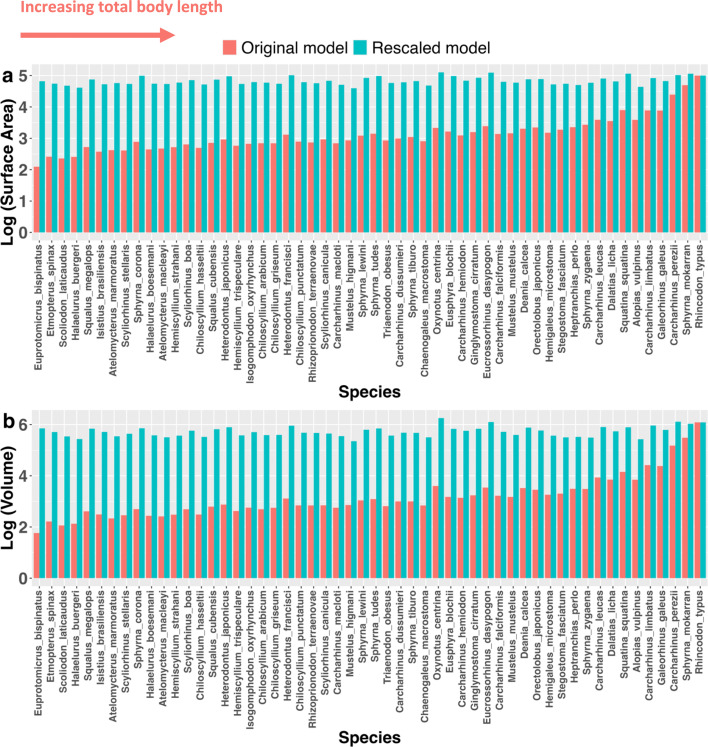
Original (orange) and rescaled (turquoise) surface area and volume values for all species included in the study. Species are ordered by the total length of original models, increasing from left to right.

Rescaled models, in which the total length of all species was altered to 500 cm, varied in surface area from 39 554 cm^2^ (*Mustelus higmani*) to 127 140 cm^2^ (*Oxynotus centrina*) and volume varied from 225 279 cm^3^ (*Mustelus higmani*) to 1 801 360 cm^3^ (*Oxynotus centrina*) (figure 2B; figure 5). This range of surface area and volume values represents only a 3.2- and 8.0-fold increase, respectively, relative to the 801- and 21 081-fold increases across original, unscaled models.

We found mixed evidence for the presence of phylogenetic signal in rescaled surface area and volume data. Both measurements exhibited some degree of phylogenetic signal (K_SA_ = 0.27, K_V_ = 0.20). In the case of surface area, this phylogenetic signal was significantly greater than expected on the basis of the null distribution of K values (*p* < 0.01); however, this was not the case for volume data (*p* = 0.07).

## 4. Discussion

Scaling ‘laws’ such as the 2/3 law underpin much of existing physiological and evolutionary theory [2,4,8–13]. How exactly complex geometric properties of organisms such as surface area and volume scale with body size can now be empirically studied at various levels of biological organization using emerging technologies and modelling approaches. We focused our analyses on extant sharks, an ancient lineage occupying a key position within the vertebrate phylogeny. Among our large dataset of extant shark species, our regression analyses reveal that deviation from expected slopes for surface area/volume relationships only occur when phylogenetic non-independence is accounted for (figure 4; table 1). In other words, deviations from expected relationships only occurred when correcting for the inherent similarity of closely related species [33]. Even then, the observed scaling coefficient of 0.64 for surface area to volume ratios deviates only 3% from the expected 0.66 coefficient (table 1). In light of the inconsistent and minor nature of these allometric deviations, and the recent trend towards prioritizing biological findings over strict statistical significance [37–39], we interpret these results as broadly complying with isometric expectations. Taking our largest (*R. typus*) and smallest (*E. bispinatus*) specimens as examples, this slight allometry only results in 11% and 6% departures from SA : V ratios expected under isometry respectively. Combined with our rescaled data (figure 5), this shows that the 2/3 law essentially holds true in this clade at the whole-organismal level, with only minor deviations. Consequently, if we eliminate differences in total length as a factor, most (but not all) of the variation in surface area and volume is removed, regardless of substantial differences in body size and shape, ecology and life history (figures 2 and 4). In the following, we discuss the ramifications of this finding and possible biological explanations for conserved surface area and volume scaling relationships.

The relatively minor degree of variation in surface area and volume values at any given body length (figures 2 and 5; table 1) and the similarity of scaling coefficients among ecotypes (figure 3; table 2) imply that in sharks, ecological selection may play a relatively minor role in determining the interspecific scaling of body dimensions. As discussed previously, metabolism and thermophysiology are some of the key factors thought to underlie the evolution of surface area and volume scaling across different levels of biological organization [2,4,8–13]. Consequently, variation in thermophysiological and ecological conditions to which cells, tissues or organisms are exposed can favour the evolution of diverse scaling relationships between surface area and volume [3]. The species included in this study exhibit a range of ecological lifestyles typified by exposure to diverse thermal environments and metabolic requirements [22,31], and it has been found that metabolism and thermal sensitivity correlate strongly with geography, activity levels and body size in sharks [40,41]. However, we found that relationships between surface area, volume and total length show little variation in relation to body size, ecological lifestyle or otherwise (tables 1 and 2; figures 3–5). Even under the simplifying assumption that all shark species are metabolically identical as they conform to the thermal conditions of their surrounding environment. This assumption remains questionable given the apparent prevalence of regional endothermy in Lamniformes [42]. Furthermore, there is no clear ecological or physiological explanation for why the scaling of surface area and volume should be conserved among sharks. True ectotherms should not be restricted to certain surface area values at the whole-organism level due to heat transfer alone. Besides physiology, locomotion and ecological lifestyle are the ecological factors most likely to influence the evolution of surface area in sharks. These factors determine the amount of lift and drag across the body surface at given swim speeds [43] and consequently the energetic cost of any given surface area to volume ratio from a locomotor perspective. The energy budget of sedentary demersal species such as heterodontiform or squatiniform sharks is unlikely to be impacted by increases in hydrodynamic drag accompanying increasing body size to the same extent as that of pelagic and highly migratory species [44]. For this reason, pelagic species are expected to have high fineness ratios (e.g. relatively long, thin body forms with elevated surface area) relative to demersal or benthic species, reducing the amount of hydrodynamic drag across the body [44,45]. However, we did not find any evidence to suggest that the surface area to volume ratio differs significantly among ecotypes (table 2). Furthermore, species differing profoundly in body form, swimming speed, swimming style and activity levels (e.g. *Squatina squatina* and *Sphyrna mokarran*) show negligible differences in surface area and volume when corrected for body size (figures 2 and 4). Hydrodynamic differences between these environments could also plausibly favour different surface area to volume scaling relationships. If hydrodynamic drag were a major determinant of surface area to volume ratios in sharks, we might expect the scaling relationship between the two variables to be steeper among pelagic taxa that are more affected by the hydrodynamic burden of increasing size. Intraspecific data from both sharks and cetaceans broadly support this concept, as active pelagic species and more sluggish, demersal species appear to differ qualitatively in the scaling of fineness ratios with total length [45,46]. However, once again, we found no evidence for hydrodynamically mediated differences in surface area to volume scaling relationships in sharks, as observed slopes did not differ between ecotypes (table 2; figure 3). These results are insufficient to fully rule out ecological selection as a causal factor underlying variation in surface area and volume, and future work considering a greater number of ecological traits and species would be necessary to do so. However, our analyses do suggest that distinct ecological lifestyles, which have been shown to correlate with other aspects of shark morphology (e.g. [47]), do not impart differential surface area to volume ratios (or scaling relationships) in sharks.

Sharks are generally thought to display phylogenetic conservatism in overall body form. Various aspects of cartilaginous fish morphology exhibit strong phylogenetic signal (e.g. [48,49]), indicating some degree of conservatism in body form evolution. Additionally, rates of molecular evolution in cartilaginous fishes appear to be lower than other taxa of comparable size [50,51]. In line with the concept of phylogenetic conservatism, we found that rescaled shark volume data exhibited strong phylogenetic signal, indicating that the interspecific distribution of body volume (when corrected for total length variation) is strongly influenced by the phylogenetic relationships among taxa. However, the same is not true of surface area, which does not show significant phylogenetic signal. This indicates that unlike volume, the surface area of sharks is not constrained by phylogenetic relationships. Thus, overall, phylogenetic conservatism is not sufficient to explain the nature of scaling trends underlying surface area to volume ratios in sharks. Additionally, even if strong phylogenetic signal were found, this would simply indicate that closely related species have similar surface area to volume ratios. It would not provide any ultimate explanation for the nature of these observed scaling relationships.

In the absence of any apparent relationship between the surface area to volume ratio and ecology or physiology (figures 3 and 4; table 2) that could hint at strong ecological selection, an intriguing possible explanation for the observed scaling trends is that surface area and volume scaling relationships are constrained developmentally (i.e. by conserved patterns of embryonic morphogenesis). Both evolutionary theory and empirical evidence suggest that developmental processes can strongly influence morphological scaling relationships [52,53], and indeed, developmental constraint is typically the favoured explanation in the absence of evidence for strong natural selection [52]. Importantly, each of the proposed mechanisms by which organisms could alter the scaling of surface area and volume (e.g. fractality, dissimilitude and internalization, *sensu* [3], would probably incur some energetic cost. In line with this possibility, previous work on embryogenesis in zebrafish has shown that alterations to surface area in the absence of any changes to volume incur measurable energetic costs [54]. It is thus likely that any significant shift in surface area at the whole-organism level, either through ontogeny or across phylogeny, would incur substantial energetic costs. There are also known spatial constraints on cell proliferation that are thought to act to control growth and maintain tissue integrity [55]. Developmental constraints on tissue allocation and organismal geometry are also well known in plants [56,57] and the gills of fishes [58,59]. Furthermore, across marine vertebrate diversity, elongate and anguilliform taxa tend to have small fins/appendages relative to body size [60–62]. This body form intrinsically elevates surface area of the trunk, which, in the presence of developmental constraint on tissue allocation, would need to be compensated for by a reduction in appendage size. It should be noted that while the relationship between body elongation and paired appendage reduction is seen across vertebrate diversity [62,63], it is not universal. For example, the longfin zebrafish mutant exhibits a considerably enlarged caudal fin surface area relative to wild type individuals, despite no substantial changes in total length [64]. The developmental basis of body elongation and appendage reduction in vertebrates is relatively well understood, with both apparently relating principally to shifts in *Hox* gene expression domains [63] and possibly the expression of *cdx* genes [62] among others. The reliance on shared developmental-regulatory machinery to modulate both trunk and appendage area [62,63] provides reasonable basis to suggest the presence of developmental constraints on tissue allocation, as selection to modify gene expression in one region of the body may have downstream consequences for other aspects of morphology. Consequently, we hypothesize that at the whole-organism level in sharks, surface area and volume are constrained to a basic allotment and spatial arrangement of tissue per unit body size, and that the energetic expenditure and cell proliferation required for any substantial deviation from the 2/3 scaling law may simply be too costly.

We acknowledge the need for additional data regarding the energetic costs of tissue allocation in sharks and other cartilaginous fishes. Existing data are drawn from a range of organisms from individual cells [3], to plants [56], squamates [63] and bony fishes [54,58,59,62]. While some bony fishes exhibit ecological similarities to some sharks, they also represent a fundamentally distinct biomechanical and physiological system to cartilaginous fishes [65]. Consequently, developmental constraints and energetic costs associated with morphogenesis in bony fishes and other vertebrates are not necessarily analogous to those in sharks and their relatives. However, the developmental-regulatory pathways underlying the embryonic patterning of the axial skeleton in cartilaginous fishes, including the importance of *Hox* domains for axial and paired fin morphology, do appear strikingly similar to those of other vertebrates (66 [67]. Ultimately, as experimental manipulation of tissue allocation was beyond the scope of this study, additional work is required to assess the metabolic costs of morphogenesis across a range of shark species.

## 5. Conclusions

In general, we found only minor deviations from the 2/3 law in sharks, hinting at a potentially universal principle governing body form and function at the whole-organism level, across a range of sizes, shapes and ecological niches. The implications of this finding extend far beyond morphological scaling. By informing our understanding of heat dissipation and nutrient uptake, the relationship between surface area and volume is of paramount importance to physiology. Ecologically, it provides an alternative lens to view interactions between organisms and their environment, particularly in the context of resource utilization and habitat preferences. The 2/3 law also has broad applications for conservation and industry, from predicting how sharks may respond behaviourally and morphologically to climate change to informing conservation efforts and the design of bio-inspired systems. However, further research is needed to uncover the underlying drivers of these scaling trends and to determine whether similar patterns exist across other vertebrate groups. Given the central role of surface area and volume in organismal function, such future studies will be essential for advancing our understanding of organismal physiology, ecology and evolution.

## Data Availability

All data and code necessary to replicate the results of this study can be accessed at the following DOI: https://doi.org/10.25903/rj1a-s123.
